# Left ventricular thrombus in a patient with cutaneous T-cell lymphoma, hypereosinophilia and *Mycoplasma pneumoniae* infection – a challenging diagnosis: a case report

**DOI:** 10.1186/s13019-014-0200-y

**Published:** 2015-02-19

**Authors:** Claudia Oeser, Martin Andreas, Claus Rath, Andreas Habertheuer, Alfred Kocher

**Affiliations:** Department of Surgery, Division of Cardiac Surgery, Vienna General Hospital, Medical University of Vienna, Währinger Gürtel 18-20, 1090 Vienna, Austria

**Keywords:** Cardiac masses, Cardiac thrombus, Left ventricular thrombus, T-cell Lymphoma, Hypereosinophilia, *Mycoplasma pneumoniae* infection, Cardiac MRI, Echocardiography

## Abstract

Differential diagnoses of cardiac masses include primary benign and malignant neoplasms, secondary neoplasms, and non-neoplastic masses, such as thrombi. Owing to different therapeutic approaches and the way these affect the prognosis, the early and correct diagnostic determination of the etiology of a cardiac mass is of utmost importance and essential for the appropriate management of patients.

We report a case of a 52-year-old woman with a left ventricular mass in the setting of a recent *Mycoplasma pneumoniae* infection and a medical history of cutaneous T-cell lymphoma and hypereosinophilia. Imaging findings were consistent with both an infiltrative process of the lymphoma and a cardiac thrombus. An estimated very high risk for embolization led to the indication for open-heart surgery for the removal of the cardiac mass. Histopathological examination confirmed the presence of a thrombus; there were no signs of malignancy. The patient was discharged 11 days after surgery in good general condition and is now in outpatient care for follow-up and further management.

This case highlights possible challenges in the diagnostic assessment of cardiac masses and their management in a patient with several underlying diseases and a complex medical history.

**Electronic supplementary material:**

The online version of this article (doi:10.1186/s13019-014-0200-y) contains supplementary material, which is available to authorized users.

## Background

Primary cardiac neoplasms are rare, with an average frequency of approximately 0.021% in autopsy series [1].

By comparison, secondary (metastatic) neoplasms are more common, found in 9.1% of patients with malignant diseases in an autopsy study [2]. Cardiac metastatic involvement is mainly observed in pleural mesotheliomas, melanomas, carcinomas of the lung, breast, and ovaries, and in lymphomyeloproliferative neoplasms [2].

The most frequently diagnosed cardiac masses are no neoplasms, but tumor-like lesions, such as cardiac thrombi. Intracardiac thrombi can mimic neoplasms, and despite modern imaging techniques differentiation can be difficult [3]. Owing to different therapeutic approaches and the way these affect the prognosis, the early and correct diagnostic determination of the etiology of a cardiac mass is of utmost importance and essential for the appropriate management of patients.

Systemic thromboembolism constitutes a much feared complication of left ventricular thrombi, especially in cases where it triggers cerebral events. Protrusion and mobility of a thrombus are indicators for an increased risk of embolism [4].

Anticoagulation therapy has been shown to be effective in reducing the risk for embolic events after thrombus formation in patients with myocardial infarction [5]. Anticoagulation therapy is currently the mainstay in the treatment of cardiac thrombi. However, because of the great variety of possible underlying causes and processes, a general therapeutic recommendation is lacking [6]. Thrombolysis [6] or surgical treatment [6,7] might be considered in high risk patients. Careful evaluation of risks and benefits is necessary for both interventions [6,7]. Operative treatment showed a trend towards a lower rate of post-treatment embolism compared with anticoagulation therapy alone [7]. This supports the assumption that surgical intervention may be beneficial in certain circumstances [6].

## Case presentation

A 52-year-old woman from Austria sought medical attention for a dry cough, severe shortness of breath, and increasing fatigue during her holiday trip to Australia. Symptoms first occurred four days before admission and about two weeks after long-haul air travel. Additionally, the patient presented with new-onset chest pain.

The patient was known to have had non-Hodgkin lymphoma (NHL), type cutaneous T-cell lymphoma (CTCL), subtype Sézary syndrome, for about three years. After histopathological confirmation of lymph node infiltration (stage IVA) one year ago, the patient received six cycles of chemotherapy with liposomal Doxorubicin. This treatment was followed by extracorporeal photophoresis; the last cycle was administered two weeks before she traveled to Australia. Additionally, interferon-alpha therapy was started. However, because of the occurrence of retinitis and vision disorders, administration was stopped two weeks before the patient’s journey. Her medical history also revealed hypereosinophilia and hyperlipidemia. At the start of the journey, the patient hadn’t been on any medication.

On first admission the patient presented with symptoms and signs of congestive heart failure. Physical examination showed hypotension and tachycardia (heart rate around 110 bpm). The patient had no fever, and oxygen saturation was within the normal range. On auscultation, no cardiac murmur was noted, but bilateral basal crackles were heard. A full blood count showed mild anemia (hemoglobin 113 g/l) and a total white cell count of 35.4×10^9^/l, with an increase in neutrophils (11.9×10^9^/l), lymphocytes (18×10^9^/l), eosinophils (4.4×10^9^/l), and basophils (0.5×10^9^/l). Further laboratory investigations revealed hypoalbuminemia (24 g/l) and raised concentrations of troponin I (0.23 μg/l), lactate dehydrogenase (319 U/l), CRP (34 mg/l), and BNP (2103 pg/ml). The troponin I level stayed on the same level for the first 24 hours after hospitalization. The following day, the troponin level was falling (0.177 μg/l). An electrocardiogram showed sinus rhythm with ST-segment depression and T-wave inversion in the inferolateral leads. Initial clinical differential diagnoses included non-ST elevation myocardial infarction (NSTEMI), viral myocarditis, and lower respiratory tract infection. The patient was started on acetylsalicylic acid and enoxaparin, as well as on benzylpenicillin and doxycycline. A chest X-ray detected bilateral interstitial edema and basal plate atelectasis. A computed tomography pulmonary angiogram (CTPA) ruled out pulmonary embolism but identified bilateral pleural effusions and multiple small non-specific nodules in both lungs, on a background of diffuse ground-glass change. Transthoracic echocardiography (TTE) found a cardiac mass located in the apical region of the left ventricle. The subjacent apical segments of the left ventricle moved poorly. Initially an infiltrative process in the endocardium and adjacent myocardium with inflammatory or malignant cells was suspected, as a result of cardiac involvement by the lymphoma. After these diagnostic findings, anticoagulation and antibiotics were stopped.

**Figure 1:**
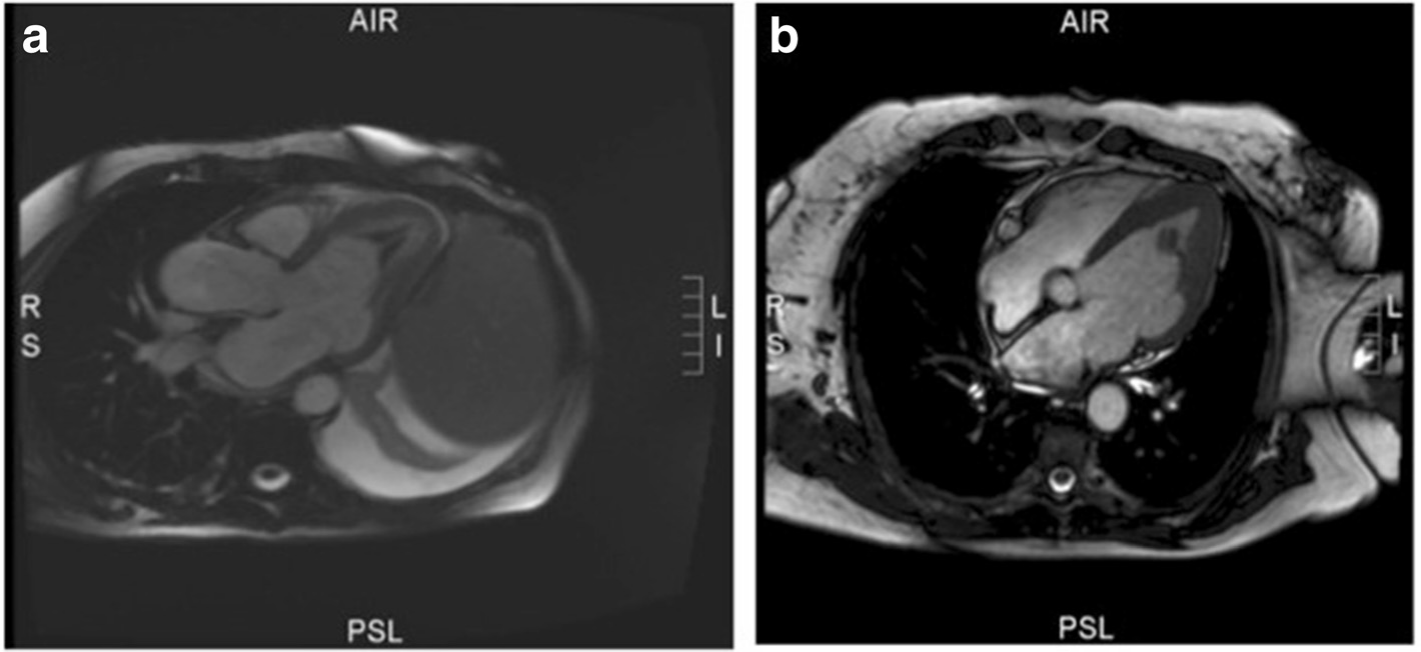
**Cardiac MRI (T2-weighted). a**: Cardiac MRI showing the thrombus filling the apex. **b**: Cardiac MRI showing the shrunken, free-floating, and pedunculated thrombus, arising from the lateral wall.

Because the need for a cardiothoracic intervention was highly probable, the patient was referred to a tertiary care center for further diagnostic work-up and treatment the day after first admission. Computed tomography (CT) scans of chest, abdomen, and pelvis showed the presence of a soft tissue mass arising from the lateral left ventricular wall, projecting into the cavity. Furthermore, CT revealed ectasia of the ascending aorta (diameter: about 40 mm), bilateral axillary and inguinal lymphadenopathy, with lymph nodes measuring up to 12 mm, and mild to moderate hepatosplenomegaly. Cardiac findings on magnetic resonance imaging (MRI) seemed consistent with endomyocardial fibrosis of the apical left ventricle, which was associated with a large concentric apical thrombus (Figure 1a). A small pericardial effusion was detected. No regional wall motion abnormality was found. Subsequently, anticoagulation was started with enoxaparin. Furthermore, the patient was treated with oral prednisolone, and she responded well to forced diuresis with furosemide. Because the positive serology test for *Mycoplasma pneumonia* indicated recent infection, the previous antibiotic treatment, consisting of piperacillin and tazobactam, was replaced by ceftriaxone and azithromycin, followed by amoxicillin in combination with clavulanic acid.

At the patient’s request, she was transferred back to Europe by air ambulance, 21 days after she was first admitted to hospital, and was hence referred to the Vienna General Hospital. The patient was admitted with mild to moderate dyspnea in a stable condition, complaining about no other symptoms. Troponin I levels were within the normal range. ECG showed sinus rhythm with ST-segment depression, and T-wave inversion in precardiac leads.

The first TTE after admission showed that the apical third of the mildly hypertrophic left ventricle was completely filled with an intracavital mass (30×34mm), which was suspected to be a thrombus with or without involvement of neoplastic cells. Akinesia of the subjacent myocardium was detected, but the ejection fraction was calculated to be 68%. An additional echocardiographic cine-loop shows this in more detail (see Additional file 1). Because scintillating scotomas occurred in the left visual field, cranial CT and MRI were performed. The latter confirmed the presence of two small point-shaped lesions, one in the right cerebellar hemisphere and one in the right precentral gyrus, consistent with a recent embolic/ischemic event. Five days after the initial TTE, cardiac MRI showed that the mass had shrunk to a considerably smaller size (diameter: approx. 10 mm), free-floating and pedunculated, arising from the lateral wall (Figure 1b). An additional echocardiographic cine-loop shows this in more detail (see Additional file 2). In combination with the diagnostic finding of the fluorine-18 fluorodeoxyglucose (F-18 FDG) positron emission tomography/CT, the suspect hypertrophic apical region seemed consistent with an infiltrative process of the lymphoma. The myocardium was thickened and there was a grayish discoloration of the tissue. The akinesia described earlier was not detectable anymore. In the repeat TTE performed one day later, the suspect hypertrophic apical region seemed consistent with endomyocardial fibrosis. Echocardiographic findings leading to the picture of endomyocardial fibrosis included the combination of myocardial thickening, a thrombus adherent to the endocardial surface, and enlargement of the left atrium. The patient received anticoagulation therapy between the imaging procedures showing reduction in size of the intracardiac mass.

**Figure 2:**
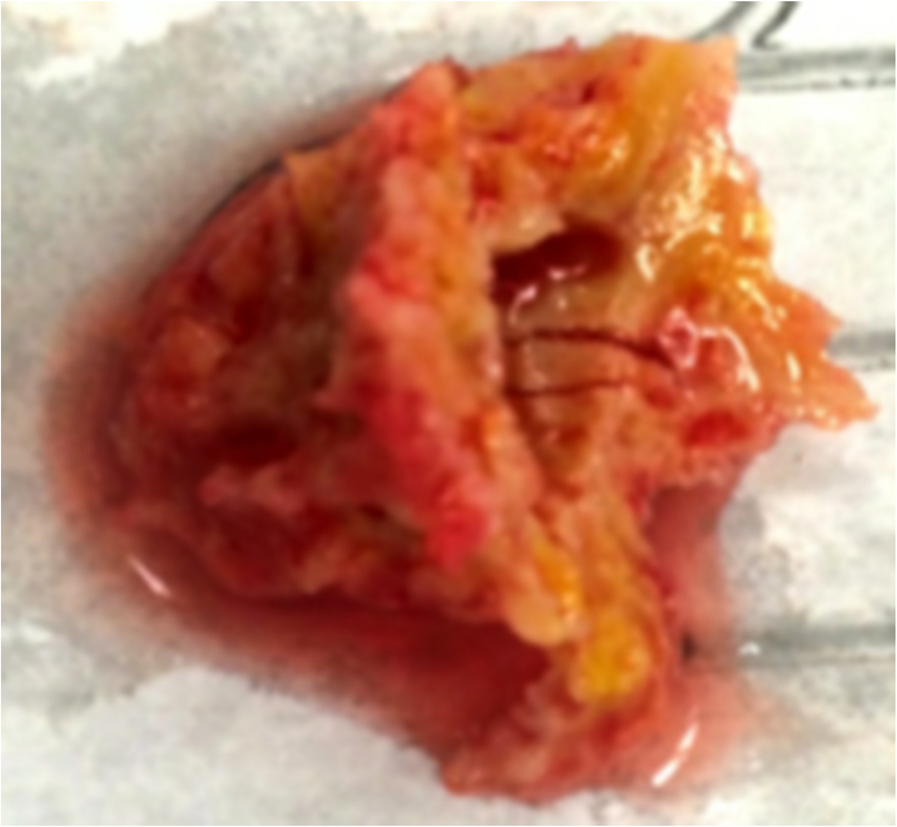
**Cardiac thrombus after surgical removal.**

The disease course with shape change and size reduction of the intracardiac mass prompted the assumption that the intracardiac mass was most likely a thrombus with an estimated very high risk for embolization. Consequently, urgent surgery was indicated. Uneventful open-heart surgery using cardiopulmonary bypass was performed through a median sternotomy. Removal of the highly mobile left intraventricular mass was easily achieved by access via the left atrium (Figure 2). Histopathological examination of this tissue showed predominantly thrombotic material consisting of layered fibrin, partly encircled by histiocytes, and additionally a collagen rich scar area. After incision of the aorta, a suspicious incisional margin involving the apical region up to the middle of the left ventricle was identified. The margin’s boundary to the surrounding tissue was not defined, and therefore it raised suspicion for being a malignant infiltration. The margin was removed as far as possible, and a biopsy of the apical region was taken. Malignant cells were not detected on histological evaluation.

Postoperatively performed echocardiography showed normal left ventricular systolic function and normal wall movement in the absence of akinetic areas. The patient made an uneventful postoperative recovery and was therefore discharged, 11 days after surgery and in good general condition.

Oral anticoagulation with phenprocoumon was initiated, and the patient is now in outpatient care for follow-up and further management.

## Conclusion

In most cases, TTE is the initial performed imaging technique for the detection and diagnosis of cardiac masses. One the one hand, TTE can provide reliable anatomic and functional features of a detected cardiac mass, but, on the other hand, its ability to characterize tissue is limited. Cardiac MRI is superior in this regard and should therefore be the diagnostic tool of choice for the assessment of cardiac masses today [3].

For example, where ventricular thrombi are concerned, sensitivity and specify in contrast-enhanced MRI were highest compared with TTE and transesophageal echocardiography (TEE). With an overall sensitivity of only 23% in TTE, cardiac MRI (overall sensitivity of 88%) may also be performed in patients considered at risk for thrombus formation without any finding on TTE [8].

In our patient, MRI was the first modality to correctly identify the cardiac mass as a thrombus. However, the suggested infiltrative process of lymphoma was not confirmed in the histopathological examination. This highlights the remaining difficulties in diagnostic MRI scans of cardiac masses. Histopathological examination is essential for the establishment of the correct diagnosis [9].

Thrombus formation in the left ventricle is typically associated with left-ventricular-wall motion disorders and impaired left ventricular function [6]. Consequently, left-ventricular thrombosis mainly occurs in coronary artery disease, especially as a complication after myocardial infarction [10], as well as in dilated cardiomyopathy [11], transient apical ballooning [12], and myocarditis [13]. Thrombophilic disorders - for example, antiphospholipid syndrome [14] - can predispose to cardiac thrombosis in the absence of heart disease. Cardiac involvement with development of intracardiac thrombosis is also described in the setting of systemic diseases, such as American trypanosomiasis [15], and amyloidosis [16].

A meta-analysis by Caruso et al. [17] showed a global thrombosis incidence of 6.4% in adult lymphoma patients. Although patients with hematologic malignancies are known to be at an increased risk for venous thrombosis, 16.5% of total thrombotic events were observed in the arterial system.

Potential mechanisms, which lead to thrombosis in hypereosinophilia, include inhibition of anticoagulant pathways, activation of coagulation and endothelial damage caused by cytotoxic effects [18].

It is assumed that an association exists between *Mycoplasma pneumoniae* infection and the occurrence of arterial and venous thrombi. The underlying pathogenesis remains unclear, however. Mechanisms of direct invasion and mechanisms of autoimmune modulation are under discussion. Cardiac thrombi in patients with *Mycoplasma pneumoniae* infection are extremely rare [19].

In conclusion, one of the patient’s underlying diseases or the combination of those could have put her in a hypercoagulable state, which resulted in thrombus formation. In hindsight, based on the clinical and diagnostic findings including transient wall motion abnormalities, moderate rise of troponin I levels declining after 24 hours and ECG changes, takotsubo cardiomyopathy could also have been a reasonable explanation for the patient’s condition. This unusual case highlights possible challenges in the diagnostic assessment of cardiac masses and their management in patients with several underlying diseases and a complex medical history.

## Consent

Written informed consent was obtained from the patient for publication of this Case report and any accompanying images. A copy of the written consent is available for review by the Editor-in-Chief of this journal.

## Additional files

Additional file 1:
**Echocardiographic cine-loop 1: apical third of the left ventricle completely filled with the thrombus and akinesia of the subjacent myocardium.**
Additional file 2:
**Echocardiographic cine-loop 2: thrombus considerably smaller in size, free-floating and pedunculated arising from the lateral wall of the left ventricle without regional akinesia.**

## Abbreviations

NHL: Non-Hodgkin lymphoma
CTCL: Cutaneous T-cell lymphoma
NSTEMI: Non-ST elevation myocardial infarction
CTPA: Computed tomography pulmonary angiogram
TTE: Transthoracic echocardiography
CT: Computed tomography
MRI: Magnetic resonance imaging
F-18 FDG: Fluorine-18 fluorodeoxyglucose
TEE: Transesophageal echocardiography
